# Role of Irisin in Myocardial Infarction, Heart Failure, and Cardiac Hypertrophy

**DOI:** 10.3390/cells10082103

**Published:** 2021-08-16

**Authors:** Ming-Yun Ho, Chao-Yung Wang

**Affiliations:** 1 Division of Cardiology, Chang Gung Memorial Hospital, Linkou Medical Center, Taoyuan 330, Taiwan; B9005017@hotmail.com; 2 School of Medicine, College of Medicine, Chang Gung University, Taoyuan 330, Taiwan; 3 Institute of Cellular and System Medicine, National Health Research Institutes, Zhunan 350, Taiwan; 4 Department of Medical Science, National Tsing Hua University, Hsinchu 300, Taiwan

**Keywords:** irisin, myocardial ischemia, myocardial infarction, heart failure, hypertension, cardiac hypertrophy

## Abstract

Irisin is a myokine derived from the cleavage of fibronectin type III domain-containing 5. Irisin regulates mitochondrial energy, glucose metabolism, fatty acid oxidation, and fat browning. Skeletal muscle and cardiomyocytes produce irisin and affect various cardiovascular functions. In the early phase of acute myocardial infarction, an increasing irisin level can reduce endothelial damage by inhibiting inflammation and oxidative stress. By contrast, higher levels of irisin in the later phase of myocardial infarction are associated with more cardiovascular events. During different stages of heart failure, irisin has various influences on mitochondrial dysfunction, oxidative stress, metabolic imbalance, energy expenditure, and heart failure prognosis. Irisin affects blood pressure and controls hypertension through modulating vasodilatation. Moreover, irisin can enhance vasoconstriction via the hypothalamus. Because of these dual effects of irisin on cardiovascular physiology, irisin can be a critical therapeutic target in cardiovascular diseases. This review focuses on the complex functions of irisin in myocardial ischemia, heart failure, and cardiac hypertrophy.

## 1. Introduction

Irisin is a myokine with 112-amino acid, glycosylated protein hormone. It is a cleavage product of the polypeptide from fibronectin type III domain-containing 5 (FNDC5), a transmembrane protein of skeletal muscle. Peroxisome proliferator-activated receptor-γ coactivator 1α (PGC-1α) mediates exercise-related effects by stimulating an increase in the expression of FNDC5 and the release of irisin. This process can activate mitochondrial energy regulation, glucose metabolism, fatty acid oxidation, and fat browning. Exercise influences various organs, such as the brain, heart, muscles, adipose tissue, and liver. Irisin is a multifunctional hormone acting on metabolism, diabetes mellitus, and cardiovascular diseases. In this regard, irisin, FNDC5, and PGC-1α are therapeutic targets of metabolic and cardiovascular disease.

Exercise increases irisin abundance, which can fine-tune body composition in individuals with obesity. Irisin regulates appetite by the expression of brain-derived neurotrophic factor (BDNF) and neuronal activity [1]. Irisin can facilitate neural differentiation through the ERK1/2 MAPK pathway [2]. Irisin can trigger adipocyte fatty acid metabolism and induce fat browning through thermogenesis and energy homeostasis [3]. Skeletal muscle secretes cytokines and peptides after exercise, including interleukin-6 (IL-6), IL-8, IL-15, fibroblast growth factor, BDNF, and irisin [4,5]. These myokines act, together with irisin, to modulate energy homeostasis and exercise effects on the cardiovascular system [6]. Irisin overexpression can increase energy expenditure and improve insulin resistance in a diabetic animal model [3]. Cardiomyocytes produce more irisin than skeletal muscle does [7]. After myocardial injury, cardiac cells begin to repair the myocardium. This reparative process is associated with oxidative stress, apoptosis, inflammation, and energy balance. Irisin plays an essential role during this recovery phase. Subsequently, regeneration of the myocardial cells is responsible for the later recovery of myocardial function. Irisin promotes cardiac progenitor cell-induced myocardial repair [8,9]. In heart failure, exercise and rehabilitation can improve long-term prognostic outcomes. Irisin can affect the outcomes of patients with heart failure by interacting with other exercise-related myokines [10]. By reducing oxidative stress, irisin improves endothelial function in patients with diabetes [11]. Through vascular endothelial nitric oxide synthase (eNOS) signaling, irisin modulates blood pressure and endothelial dysfunction [12]. Irisin also acts as a regulator of macrophages and affects atherosclerosis and host defense [13].

**Figure 1:**
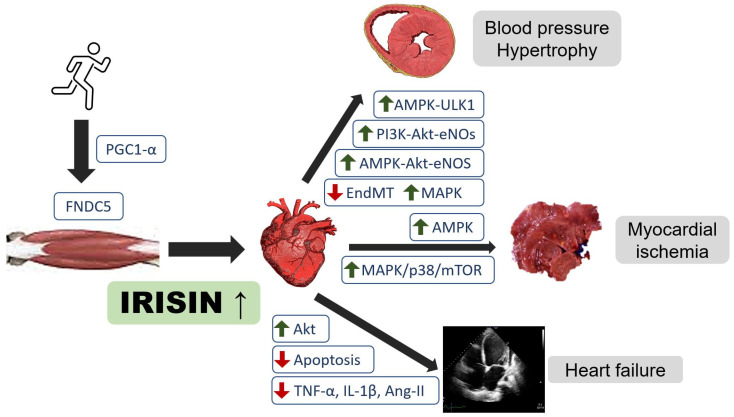
Regulatory role of irisin in heart failure, cardiovascular disease, and myocardial ischemia. Akt, protein kinase B; AMPK, adenosine monophosphate-activated protein kinase; eNOS, endothelial nitroxide synthase; PGC-1α, peroxisome proliferator-activated receptor-γ coactivator-1α; ULK1, serine/threonine-protein kinase; EndMT, endothelial-to-mesenchymal transition; MAPK, mitogen-activated protein kinase; mTOR, mechanistic target of rapamycin.

However, many irisin studies have shown conflicting results between irisin levels and associated prognostic value in cardiovascular diseases [10,14,15,16]. The role of irisin may be different in different phases of cardiovascular diseases (Figure 1). This review aims to provide an overview of the association between irisin and cardiovascular function and the potential role of irisin in cardiovascular diseases.

## 2. Acute Myocardial Infarction

Acute myocardial infarction is the leading cause of death and morbidity in cardiovascular diseases [17]. Myocardial infarction is caused by progressively atherosclerotic coronary arteries and ruptured lipid plaques, which result in blood flow occlusion and myocardial ischemia. Circulating irisin is associated with atherosclerosis and acute myocardial infarction [14,18]. Patients with stable coronary artery disease and more advanced lesions have lower serum irisin [19]. The pooled data in a meta-analysis from 2000 to 2017 showed that irisin levels were lower in patients with cardiovascular disease than in healthy controls [16]. Irisin levels were significantly higher in patients with diabetes without coronary artery disease than those with diabetes and coronary artery disease [15]. However, in some studies, a higher irisin level was associated with an increased risk of coronary artery disease, acute coronary syndrome, and heart failure [14,20]. The exact link between irisin abundance in patients and the development of cardiovascular disease is still under investigation.

In mice, irisin suppresses neointima formation and attenuates the development of atherosclerotic aortic lesions [21]. Irisin also inhibits atherosclerosis by promoting endothelial proliferation [22]. Recent data also showed that irisin inhibits hepatic cholesterol synthesis [23]. Furthermore, the farnesoid X receptor, which is a ligand-activated transcription factor in cholesterol metabolism, regulates reverse cholesterol transport and cholesterol homeostasis through irisin transcription [24].

Irisin is highly expressed in the myocardium. Irisin abundance is significantly correlated with different stages after myocardial infarction and cardiac repair [14,25,26]. Cardiac repair after myocardial infarction includes several phases [27,28]. The initial step is mediated by inflammation and immune cell migration. In this phase, the immune cells begin to digest the debris of cell death and extracellular tissue. After 3 to 5 days, the reparative phase initiates simultaneously with fibrosis formation and neovascularization. In this stage, energy metabolism through the AMPK pathway with macrophage migration is essential to achieve an optimal repair process [29,30]. If the inflammatory phase is prolonged, the residual dead cells and tissue will influence further regeneration, causing poor cardiac remodeling and infarct enlargement. Heart function decrease leads to chamber dilatation and systolic myocardial dysfunction [28]. Irisin plays a significant role in these stages of myocardial infarction.

### 2.1. Inflammatory Phase after Myocardial Infarction

In the acute phase of myocardial infarction, coronary blood supply, and myocardial demand imbalance cause cardiomyocyte damage and energy depletion. Therefore, decreasing cell damage and improving cardiac function are essential for cardiomyocyte survival. The myocardial injury, including mitochondrial dysfunction and reactive oxygen species (ROS) influences the myocardium repair and remodeling processes. Irisin is critical in mitochondrial homeostasis in myocardial infarction. After an ischemic injury, irisin interacts with the mitochondrial uncoupling protein [31,32], preventing mitochondrial dysfunction and reducing oxidative stress.

In early-stage ischemia, hypoxemia impairs endothelial cell function to increase permeability and leukocyte infiltration [27]. Autophagy, apoptosis, and necrosis are involved in the pathogenesis of cardiomyocyte death [33]. Clearance of apoptotic neutrophils, recruitment of inhibitory monocyte subsets and regulatory T cells, macrophage differentiation, may play a role in post-infarction inflammation [34]. After reperfusion, ROS are generated, and the complement pathway is activated. These signals activate inflammatory cytokines, including tumor necrosis factor-α, IL-1β, IL-6, IL-18, and infiltrating leukocytes and macrophages. Injection of irisin can protect heart against ischemia and reperfusion injury through mitochondrial function improvement [35]. Irisin can attenuate active caspase-3 and cleaved poly(ADP-ribose) polymerase and increase hypoxia resistance in cardiomyoblasts to suppress mitochondrial apoptosis and swelling [31]. Two studies demonstrated that irisin attenuates hypoxic injury in diabetic mice through the adenosine monophosphate-activated protein kinase (AMPK) pathway to improve mitochondrial function [36,37]. Irisin functions through a superoxide dismutase 2-dependent mitochondrial mechanism to protect against ischemia-reperfusion injury [38]. Irisin activates dynamin GTPase Opa1-induced mitophagy to protect cardiomyocytes against apoptosis after myocardial infarction [39]. Irisin also decreases ischemic injury through a mitochondrial ubiquitin ligase-dependent mechanism and relieves endoplasmic reticulum stress [40].

The abundance of irisin changes dynamically after acute myocardial infarction. Irisin increases after 8 h of myocardial infarction, then gradually decreases on day three in patients with acute ST-segment elevation myocardial infarction [14]. This pattern of irisin release after myocardial infarction was confirmed in another study in serum and saliva [41]. In the early phase of myocardial infarction, higher irisin levels may decrease injury to protect against ischemic change. Irisin reduces endothelial damage by inhibiting inflammation and oxidative stress [42].

### 2.2. Reparative and Proliferative Phase after Myocardial Infarction

In the late phase of myocardial infarction, the inflammatory response is tapered and resolves after 3 to 5 days. The myocardium begins the repair process, which necessitates cell remodeling and ROS production. Immune regulation and myofibroblasts are activated for the repair mechanisms. These processes contribute to neovascularization, scar formation, and cardiac repair. The immunomodulation and anti-inflammatory effects last up to 14 days. The cardiomyocytes secrete several paracrine signals, including islet-derived 3β, to regulate macrophages and inhibit inflammation [43]. Depletion of irisin in mice increases the pro-inflammatory cytokines, including increasing levels of IL-6 and TNF-α and decreasing level of IL-10 [44].

A clinical study extensively examined the level of irisin 1 month after myocardial infarction [14]. The myocardium is in the repair and proliferative stages 1 month after myocardial infarction. The irisin level in this stage reflects its association with myocardial repair. In contrast to the beneficial effects of irisin in acute stages, higher serum levels of irisin in patients with myocardial infarction in this stage are associated with more heart failure events. Patients with the highest levels of irisin have an increased risk of major adverse cardiovascular events [14]. Several mechanisms underlie these contradictory results in the acute stages versus chronic stages after myocardial infarction. Forced overexpression of irisin in mice increases mitochondrial respiration and generates excessive ROS. Treating cardiomyocytes with higher irisin levels increases cleaved caspase-9 and hypoxia-induced apoptosis [32]. Patients with myocardial infarction with higher irisin levels may have incomplete inflammation resolution. The large infarct area of the myocardium results in more inflammatory activation and leads to progressive chamber dilatation and heart failure. The inflammatory responses after acute myocardial infarction sustain to the reparative and proliferative phase. This subsequent inflammatory reaction interacts with the reparative and proliferative mechanisms. This incomplete anti-inflammatory response is associated with poor cardiovascular outcomes. The higher level of irisin may be secondary to these prolonged inflammatory reactions [14].

**Figure 2:**
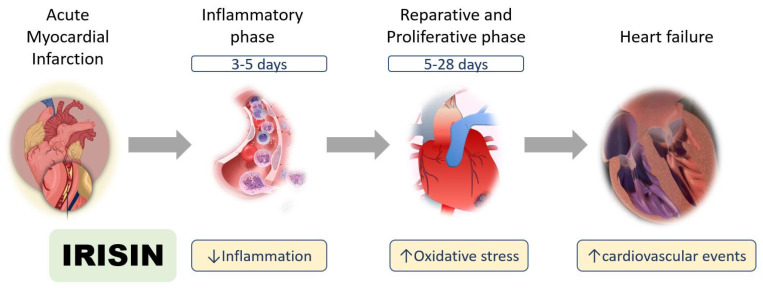
Roles of irisin in myocardial ischemia. Irisin has multiple functions after myocardial infarction in the inflammatory, reparative, and proliferative phases.

In short, different mechanisms may underlie irisin abundance after myocardial infarction (Figure 2). More evidence and research are warranted to identify the complex irisin reactions after myocardial injury. In the future, irisin may be a therapeutic target to improve myocardial injury after myocardial ischemia and enhance cardiac repair.

## 3. Heart Failure

### 3.1. Oxidative Stress and Mitochondria Dysfunction in Heart Failure

Heart failure is the terminal stage of many cardiovascular diseases with high mortality [45]. The molecular mechanisms of heart failure are associated with oxidative stress, mitochondrial dysfunction, energy imbalance, and deranged substrate use [46]. Cardiomyocytes are highly metabolically active, with mitochondria as their primary energy source [47]. If mitochondrial energy production is deranged, the energy insufficiency results in heart failure progression. Clinical data have shown that patients with heart failure have abnormal mitochondrial energetics, including reduced energy reserves, impaired fatty acid oxidation, and decreased peripheral oxygen extraction [46,48].

The mitochondria in cardiomyocytes are dynamic and responsive to various physiological or pathological conditions [49]. Exercise training leads to increases in mitochondrial mass and mitochondrial biogenesis [50]. Exercise affects intracellular calcium, increases cellular adenosine triphosphate turnover, enhances the ratio of nicotinamide adenine dinucleotide (NAD^+^) to NAD + hydrogen, and increases ROS [51]. All this signaling activates PGC-1α [52]. PGC-1α is the master regulator of mitochondrial biogenesis and plays a vital role in regulating cardiac function [53]. Fine-tuning PGC-1α expression maintains the cardiac physiology and the balance of cardiac energy biogenesis of mitochondria. In contrast, heart failure is associated with disordered mitochondrial structure and a drop in mitochondrial oxidative capacity [46,48]. Pathological remodeling of the heart, usually due to ischemia with myocardium loss, results in the mismatch of mitochondrial energy demand and generation [46,48]. Heart failure is associated with the downregulation of transcription factors of mitochondrial biogenesis through PGC-1α [54,55]. Decreased mitochondrial density and maximal oxygen uptake occur at the late stage of heart failure in patients [48]. Moreover, the polymorphisms in the PGC-1α gene are correlated with increased risks of hypertrophic cardiomyopathy and heart failure [56]. PGC-1α is required to induce ROS-detoxifying enzymes, such as glutathione peroxidase 1 and superoxide dismutase 2. PGC-1α is a critical regulator of mitochondrial ROS metabolism in cardiac function, providing a potential target for heart failure treatment.

Irisin was identified as a PGC-1α–dependent myokine [3]. Irisin plays an essential role in energy homeostasis through white fat browning and muscle fiber type switching [57]. Irisin can modulate glucose homeostasis by adipocytes via UCP1 expression and liver via gluconeogenesis [58]. Irisin can ameliorate lipid metabolic derangements in obesity and enhance lipolysis via the cAMP-PKA-HSL pathway [59,60], and irisin is secreted from skeletal muscle and adipocytes after exercise [61]. In cardiomyocytes, *FNDC5* mRNA expression and irisin are abundant and associated with high energy expenditure [7,32,62]. Exercise increases cardiac and plasma levels of irisin in rats [63]. Increases in irisin also improve cardiac progenitor cell-related cardiac repair [8,9]. However, we will need more evidence to confirm the effects of irisin on cardiac repair. In a rat model of chronic heart failure, the circulating irisin level was reduced [64]. These exercise-related irisin enhancements of heart function have age-dependent effects, which might be crucial for aging patients with heart failure.

Irisin could improve cardiac remodeling by inhibiting oxidative stress and attenuating Akt signaling activation [65,66]. In rats with ischemic cardiomyopathy and heart failure, the decreased irisin level was modulated by inflammatory cytokines and angiotensin II [64]. In cardiomyocytes after angiotensin II injury, irisin can lessen apoptosis through autophagy [67]. As demonstrated by the link between energy metabolism and irisin, irisin might be essential in heart failure.

### 3.2. Irisin in Patients with Heart Failure

The first study in patients with heart failure revealed high *PGC-1α* and *FNDC5* expression from skeletal muscle biopsy samples correlation with a better functional capacity [68]. High aerobic cardiopulmonary exercise can improve FNDC5 expression in patients with heart failure [68]. In patients with acute heart failure, serum irisin predicted 1-year mortality. A higher level of irisin in patients with heart failure was associated with more deaths than a lower level of irisin [10]. Cachexia is an unfavorable outcome of heart failure. Cachexia is caused by muscle wasting and the exacerbation of heart failure decompensation. A low level of circulating irisin was noted in female patients with heart failure with cachexia [69]. By contrast, another study showed a high level of irisin in heart failure with cachexia [70].

Heart failure with a preserved ejection fraction has a more complex mechanism than that with a reduced ejection fraction does. It affects both systolic and diastolic function of the heart and is related to cardiac hypertrophy and myocardial fibrosis [71]. The traditional treatment, including β-blockers and angiotensin-converting enzyme inhibitors, was ineffective in heart failure with preserved ejection fraction. One small study revealed that irisin levels were higher in patients with heart failure with preserved ejection fraction than those with reduced ejection fraction [72].

To date, research in irisin and heart failure is still insufficient. Whether irisin is a biomarker for heart failure or the exact mechanistic signaling of heart failure is unclear. Heart failure has different stages, including acute heart failure, chronic heart failure, or chronic heart failure with acute decompensation. In the different stages of heart failure, the role of irisin might have multiple functions. In acute ischemic injury with heart failure, higher irisin could waste more energy and increase oxidative stress and ROS [32]. A higher level of irisin is associated with higher mortality in acute heart failure [10]. The higher irisin level associated with greater energy expenditure may lead to heart failure exacerbation and higher mortality. Higher irisin might enhance the energy demand and produce more oxidative stress. In the chronic heart failure stage, lower irisin might represent muscle loss and cachexia [69]. However, we need a large cohort study to prove this observation in the future. The oxidative stress and increased mitochondrial respiration due to irisin overexpression and impaired mitochondrial biogenesis could lead to heart failure progression and cardiac fibrosis [32,48]. Current therapeutic goals in patients with heart failure are neuroendocrine activation inhibition, ventricular unloading, and heart rate reduction. However, no therapeutic medication can directly target metabolic mechanisms or mitochondrial biogenesis. Irisin might be a new target of heart failure treatment in metabolic and energy regulation.

## 4. Hypertension and Ventricular Hypertrophy

Hypertension is the most prevalent cardiovascular disease [73]. Uncontrolled hypertension increases several other cardiovascular risks [74]. Exercise is a lifestyle modification used in the treatment of hypertension [75]. However, the mechanism linking exercise and blood pressure is not fully understood. Irisin abundance is increased with exercise, and regular exercise can decrease blood pressure as a lifestyle modification in people with hypertension [3,4,75]. Multiple studies have revealed an association between irisin levels and blood pressure. Systolic and diastolic blood pressure were positively correlated with irisin concentration in patients without hypertension [76]. In patients with preeclampsia, irisin expression has a negative correlation with blood pressure, but *FNDC5* expression in the placenta has a positive correlation with blood pressure [77]. In patients receiving antihypertensive medication, the levels of irisin are increased [78]. In hypertensive rats, injection of irisin can increase blood pressure through hypothalamic paraventricular nucleus neuron activation and reduce blood pressure by mesenteric artery dilatation through endothelium-dependent and endothelium-independent mechanisms [79,80]. Irisin has no direct vasorelaxant effect on mesenteric arteries in rats. However, irisin can activate AMPK. The activated AMPK then phosphorylates Akt and eNOS with increased NO production [12,81]. In obese mice, irisin improves endothelial function through the AMPK/eNOS pathway [82]. Irisin promotes endothelial function by activating the ERK signaling pathway. Irisin treatment increases endothelial cell proliferation and protects cells from high glucose-induced apoptosis through Bcl-2 and Bax expression [83].

Hypertension can influence cardiac function. High blood pressure causes pressure overload and myocardial hypertrophy, and a hypertrophic heart leads to heart failure [84]. This mechanical stress may trigger paracrine or autocrine secretion related to irisin [85]. Irisin protects against pressure overload-induced cardiac hypertrophy by inducing protective autophagy and autophagic flux by activating AMPK-ULK1 signaling [86]. Injection of irisin into hypertensive rats lowers blood pressure by reducing oxidative stress and inflammatory response. The possible mechanism was nuclear factor E2-related factor-2 (Nrf2) signaling and the hypothalamic paraventricular nucleus as sympathetic regulation [87]. A higher irisin level in patients with idiopathic pulmonary arterial hypertension reflects high pulmonary artery pressure and poor prognosis [88].

In patients with diabetes, the risks of coronary artery disease, hypertension, and myocardial infarction are increased [89]. The term ‘diabetic cardiomyopathy’ refers to myocardial dysfunction in patients with diabetes mellitus. In myocardial remodeling in mice with streptozotocin-induced diabetes, irisin has opposite effects at different doses. Low-dose (0.5 μg/g) irisin improves diabetic cardiomyopathy by inhibiting the high glucose-induced endothelial-to-mesenchymal transition by increasing Smad7 expression and suppressing the phosphorylation of Smad2 and Smad3 in human endothelial cells [90]. However, high-dose (1.5 μg/g) irisin fails to prevent ventricular dysfunction and increases collagen deposition in the heart [90]. Intraperitoneal injection of irisin in diabetic mice can increase circulating endothelial progenitor cells by activating the phosphoinositide 3-kinase/Akt pathway and eNOS expression [9].

Based on current studies of irisin, blood pressure, and cardiac hypertrophy, many signaling pathways were associated with irisin in cardiovascular effects. (Table 1) From a clinical perspective, lower serum irisin levels and higher serum irisin levels in patients sometimes have opposite effects [10,14,15,16,19]. The injection of different doses of irisin can also induce conflicting effects [62,79,80,87,90]. This suggests that irisin plays a critical role in blood pressure control, and a balanced serum level is essential in maintaining vascular tone. Another explanation is that irisin can act as a cofactor to modulate vascular function with heart hypertrophy, and irisin imbalance can lead to dysregulated cardiac physiology.

## 5. Perspectives and Conclusions

Irisin has multiple functions in diabetes, obesity, and cardiovascular disease. Irisin induces the browning of adipose tissue to promote energy expenditure which prevents obesity and metabolic syndrome [57]. Exercise adapts muscle to express more irisin in peripheral myelin nerve sheath and plays roles in neural differentiation [2,7]. It is involved in glucose metabolism, endothelial function, and neuromuscular linkage. The multiorgan function of irisin indicates that it is a double-edged sword in cardiovascular disease treatment. In blood pressure control, irisin might contribute to vasoconstriction or vasorelaxation. In the early phase of myocardial ischemia, irisin can reduce myocardial ischemic injury through mitochondria by affecting oxygen stress and apoptosis. Thus, irisin may have a protective effect during acute hypoxemia. In the late phase of myocardial infarction, higher irisin levels cause more major cardiovascular events. The overexpression of irisin also enhances cardiac cell damage. A possible mechanism is that higher irisin overdrives mitochondria to enhance oxidative stress and apoptosis, thereby influencing myocardial cell repair.

The function of irisin in the cardiovascular field is gaining interest, and many laboratories have been investigating the therapeutic roles of irisin. The following areas could be further investigated:

1. The adequate therapeutic level of irisin in myocardial infarction
2. The optimal timing of irisin administration during heart failure
3. The role of irisin as a biomarker in acute myocardial infarction and heart failure
4. The mechanism of irisin during heart failure repair (trigger or consequence)
5. The further response of irisin in vascular disease and hypertension

Further research is warranted to identify more of the essential mechanisms and therapeutic effects of irisin in cardiovascular disease. The disparities in irisin study results indicate the unknown aspects of irisin on the cardiomyocyte. With more evidence, irisin might be found to have an essential regulatory role in myocardial infarction, heart failure, and hypertension.

## Figures and Tables

**Table 1:** Signaling pathways of irisin in cardiovascular effects. TAC, transverse aortic constriction; Nrf2, E2-related factor-2; STZ, streptozotocin; EndMT, endothelial-to-mesenchymal transition; MAPK, mitogen-activated protein kinase; AMPK, adenosine monophosphate-activated protein kinase; mTOR, mechanistic target of rapamycin; ULK1, serine/threonine-protein kinase.

Signaling Pathway	Irisin Source/Dose	Target Cells/Tissues	Animal Model	Cardiovascular Effect	References
PI3K/Akt/eNOS	Recombinant Irisin 0.5 mg/kg	Blood/Bone marrow Endothelial progenitor cell	STZ-induced Diabetic Mice	Improved the function of endothelial progenitor cells	[9]
AMPK-Akt-eNOS-NO	Recombinant Irisin 0.1, 1, 10 µg/kg	Human coronary endothelial cell	Spontaneously hypertensive rats	Lowers blood pressure	[12]
MAPK/p38	Recombinant Irisin 100 mg/kg	Cardiomyocyte	Ischemia/Reperfusion Mice	Protect the heart against ischemia and reperfusion injury	[35]
AMPK/mTOR	Irisin to cell	Cardiomyocyte	High glucose-induced cardiomyocytes of rats	Ameliorates high glucose-induced cardiomyocytes injury	[36]
AMPK	Recombinant Irisin 0.5 µg/g	Cardiomyocyte	High fat diet-induced Diabetic Mice with ischemia/reperfusion	Attenuates myocardial ischemia/reperfusion injury and improves mitochondrial function	[37]
Akt	Recombinant Irisin 10 µg/kg	Cardiomyocyte	TAC-induced cardiac hypertrophic rat	Improve cardiac remodeling	[65]
MicroRNA-19b/AKT/mTOR	Irisin to cell	Oxidative stress-induced injury rat cardiac myoblast cell		Attenuates H_2_O_2_-induced apoptosis	[66]
Angiotensin II	Irisin transgenic mice	Cardiomyocyte	TAC-induced cardiac hypertrophic mice	Ameliorates apoptosis through autophagy	[67]
AMPK-ULK1	Recombinant Irisin	Cardiomyocyte	TAC-induced Cardiac hypertrophic Mice	Inducing protective autophagy and improves cardiac hypertrophy	[68]
Nrf2	Recombinant Irisin 10 µg/kg	Hypothalamic paraventricular nucleus	Spontaneously hypertensive rats	Lowers blood pressure	[87]
EndMT MAPK	Recombinant Irisin 0.5, 1.5 µg/g	Cardiomyocyte	STZ-induced Diabetic Mice	Dose-dependent bidirectional effect on myocardial fibrosis	[90]

## References

[1] Ferrer-Martínez A., Ruiz-Lozano P., Chien K.R. (2002). Mouse PeP: A novel peroxisomal protein linked to myoblast differentiation and development. Dev. Dyn..

[2] Farahabadi S.H., Ghaedi K., Zadegan F.G., Karbalaie K., Rabiee F., Nematollahi M., Baharvand H., Nasr-Esfahani M.H. (2015). Erk1/2 Is a Key Regulator of Fndc5 and Pgc1alpha Expression during Neural Differentiation of Mescs. Neuroscience.

[3] Boström P., Wu J., Jedrychowski M.P., Korde A., Ye L., Lo J.C., Rasbach K.A., Boström E.A., Choi J.H., Long J.Z. (2012). A Pgc1-Alpha-Dependent Myokine That Drives Brown-Fat-Like Development of White Fat and Thermogenesis. Nature.

[4] Pedersen B.K., Febbraio M.A. (2012). Muscles, exercise and obesity: Skeletal muscle as a secretory organ. Nat. Rev. Endocrinol..

[5] So B., Kim H.-J., Kim J.-S., Song W. (2014). Exercise-induced myokines in health and metabolic diseases. Integr. Med. Res..

[6] Brenmoehl J., Albrecht E., Komolka K., Schering L., Langhammer M., Hoeflich A., Maak S. (2014). Irisin Is Elevated in Skeletal Muscle and Serum of Mice Immediately after Acute Exercise. Int. J. Biol. Sci..

[7] Aydin S., Kuloglu T., Ogeturk M., Dabak O., Aydin S., Eren M.N., Celik A., Yilmaz M., Kalayci M., Sahin I. (2014). Cardiac, skeletal muscle and serum irisin responses to with or without water exercise in young and old male rats: Cardiac muscle produces more irisin than skeletal muscle. Peptides.

[8] Zhao Y.T., Wang J., Yano N., Zhang L.X., Wang H., Zhang S., Qin G., Dubielecka P.M., Zhuang S., Liu P.Y. (2019). Irisin promotes cardiac progenitor cell-induced myocardial repair and functional improvement in infarcted heart. J. Cell. Physiol..

[9] Zhu G., Wang J., Song M., Zhou F., Fu D., Ruan G., Zhu X., Bai Y., Huang L., Pang R. (2016). Irisin Increased the Number and Improved the Function of Endothelial Progenitor Cells in Diabetes Mellitus Mice. J. Cardiovasc. Pharmacol..

[10] Shen S., Gao R., Bei Y., Li J., Zhang H., Zhou Y., Yao W., Xu D., Zhou F., Jin M. (2017). Serum Irisin Predicts Mortality Risk in Acute Heart Failure Patients. Cell. Physiol. Biochem..

[11] Xiang L., Xiang G., Yue L., Zhang J., Zhao L. (2014). Circulating irisin levels are positively associated with endothelium-dependent vasodilation in newly diagnosed type 2 diabetic patients without clinical angiopathy. Atherosclerosis.

[12] Fu J., Han Y., Wang J., Liu Y., Zheng S., Zhou L., Jose P.A., Zeng C. (2016). Irisin Lowers Blood Pressure by Improvement of Endothelial Dysfunction Via Ampk-Akt-Enos-No Pathway in the Spontaneously Hypertensive Rat. J. Am. Heart Assoc..

[13] Mazur-Bialy A.I. (2017). Irisin acts as a regulator of macrophages host defense. Life Sci..

[14] Hsieh I.-C., Ho M.-Y., Wen M.-S., Chen C.-C., Hsieh M.-J., Lin C.-P., Yeh J.-K., Tsai M.-L., Yang C.-H., Wu V.C.-C. (2018). Serum irisin levels are associated with adverse cardiovascular outcomes in patients with acute myocardial infarction. Int. J. Cardiol..

[15] Khorasani Z.M., Bagheri R.K., Yaghoubi M.A., Chobkar S., Aghaee M.A., Abbaszadegan M.R., Sahebkar A. (2019). The association between serum irisin levels and cardiovascular disease in diabetic patients. Diabetes Metab. Syndr. Clin. Res. Rev..

[16] Guo W., Zhang B., Wang X. (2020). Lower Irisin Levels in Coronary Artery Disease: A Meta-Analysis. Minerva Endocrinol..

[17] Benjamin E.J., Muntner P., Alonso A., Bittencourt M.S., Callaway C.W., Carson A.P., Chamberlain A.M., Chang A.R., Cheng S., Das S.R. (2019). Heart disease and stroke statistics—2019 update: A report from the American heart association. Circulation.

[18] Sesti G., Andreozzi F., Fiorentino T.V., Mannino G.C., Sciacqua A., Marini M.A., Perticone F. (2014). High circulating irisin levels are associated with insulin resistance and vascular atherosclerosis in a cohort of nondiabetic adult subjects. Acta Diabetol..

[19] Efe T.H., Açar B., Ertem A.G., Yayla K.G., Algül E., Yayla Ç., Ünal S., Bilgin M., Çimen T., Kirbaş Ö. (2017). Serum Irisin Level Can Predict the Severity of Coronary Artery Disease in Patients with Stable Angina. Korean Circ. J..

[20] Aronis K.N., Moreno M., Polyzos S.A., Moreno-Navarrete J.M., Ricart W., Delgado E., De La Hera J., Sahin-Efe A., Chamberland J.P., Berman R. (2015). Circulating Irisin Levels and Coronary Heart Disease: Association with Future Acute Coronary Syndrome and Major Adverse Cardiovascular Events. Int. J. Obes..

[21] Zhang Y., Mu Q., Shao L., Wang X., Li S., Yang L., Wu Q., Zhang M., Tang D., Zhou Z. (2016). Protective Effect of Irisin on Atherosclerosis via Suppressing Oxidized Low Density Lipoprotein Induced Vascular Inflammation and Endothelial Dysfunction. PLoS ONE.

[22] Zhang Y., Song H., Zhang Y., Wu F., Mu Q., Jiang M., Wang F., Zhang W., Li L., Shao L. (2016). Irisin Inhibits Atherosclerosis by Promoting Endothelial Proliferation Through microRNA126-5p. J. Am. Heart Assoc..

[23] Tang H., Yu R., Liu S., Huwatibieke B., Li Z., Zhang W. (2016). Irisin Inhibits Hepatic Cholesterol Synthesis via AMPK-SREBP2 Signaling. EBioMedicine.

[24] Li H., Shen J., Wu T., Kuang J., Liu Q., Cheng S., Pu S., Chen L., Li R., Li Y. (2019). Irisin Is Controlled by Farnesoid X Receptor and Regulates Cholesterol Homeostasis. Front. Pharmacol..

[25] Bashar S.M., El-Sherbeiny S.M.S., Boraie M.Z. (2018). Correlation between the blood level of irisin and the severity of acute myocardial infarction in exercise-trained rats. J. Basic Clin. Physiol. Pharmacol..

[26] El-Mottaleb N.A.A., Galal H.M., El Maghraby K.M., Gadallah A.I. (2019). Serum irisin level in myocardial infarction patients with or without heart failure. Can. J. Physiol. Pharmacol..

[27] Eltzschig H.K., Eckle T. (2011). Ischemia and reperfusion—From mechanism to translation. Nat. Med..

[28] Prabhu S.D., Frangogiannis N.G. (2016). The Biological Basis for Cardiac Repair after Myocardial infarction: From Inflammation to Fibrosis. Circ. Res..

[29] Miller E.J., Li J., Leng L., McDonald C., Atsumi T., Bucala R., Young L.H. (2008). Macrophage migration inhibitory factor stimulates AMP-activated protein kinase in the ischaemic heart. Nat. Cell Biol..

[30] Qi D., Hu X., Wu X., Merk M., Leng L., Bucala R., Young L.H. (2009). Cardiac macrophage migration inhibitory factor inhibits JNK pathway activation and injury during ischemia/reperfusion. J. Clin. Investig..

[31] Chen K., Xu Z., Liu Y., Wang Z., Li Y., Xu X., Chen C., Xia T., Liao Q., Yao Y. (2017). Irisin protects mitochondria function during pulmonary ischemia/reperfusion injury. Sci. Transl. Med..

[32] Ho M.-Y., Wen M.-S., Yeh J.-K., Hsieh I.-C., Chen C.-C., Hsieh M.-J., Tsai M.-L., Yang C.-H., Wu V.C.-C., Hung K.-C. (2018). Excessive irisin increases oxidative stress and apoptosis in murine heart. Biochem. Biophys. Res. Commun..

[33] Chiong M., Wang Z., Pedrozo Z., Cao D.J., Troncoso R., Ibacache M., Criollo A., Nemchenko A., Hill J.A., Lavandero S. (2011). Cardiomyocyte death: Mechanisms and translational implications. Cell Death Dis..

[34] Frangogiannis N.G. (2012). Regulation of the Inflammatory Response in Cardiac Repair. Circ. Res..

[35] Wang H., Zhao Y.T., Zhang S., Dubielecka P.M., Du J., Yano N., Chin Y.E., Zhuang S., Qin G., Zhao T.C. (2017). Irisin plays a pivotal role to protect the heart against ischemia and reperfusion injury. J. Cell. Physiol..

[36] Deng J., Zhang N., Chen F., Yang C., Ning H., Xiao C., Sun K., Liu Y., Yang M., Hu T. (2020). Irisin ameliorates high glucose-induced cardiomyocytes injury via AMPK/mTOR signal pathway. Cell Biol. Int..

[37] Xin C., Zhang Z., Gao G., Ding L., Yang C., Wang C., Liu Y., Guo Y., Yang X., Zhang L. (2020). Irisin Attenuates Myocardial Ischemia/Reperfusion Injury and Improves Mitochondrial Function through AMPK Pathway in Diabetic Mice. Front. Pharmacol..

[38] Wang Z., Chen K., Han Y., Zhu H., Zhou X., Tan T., Zeng J., Zhang J., Liu Y., Li Y. (2018). Irisin Protects Heart Against Ischemia-Reperfusion Injury Through a SOD2-Dependent Mitochondria Mechanism. J. Cardiovasc. Pharmacol..

[39] He W., Wang P., Chen Q., Li C. (2020). Exercise enhances mitochondrial fission and mitophagy to improve myopathy following critical limb ischemia in elderly mice via the PGC1a/FNDC5/irisin pathway. Skelet. Muscle.

[40] Lu L., Ma J., Tang J., Liu Y., Zheng Q., Chen S., Gao E., Ren J., Yang L., Yang J. (2020). Irisin attenuates myocardial ischemia/reperfusion-induced cardiac dysfunction by regulating ER-mitochondria interaction through a mitochondrial ubiquitin ligase-dependent mechanism. Clin. Transl. Med..

[41] Aydin S., Aydin S., Baydas A., Kobat M.A., Kalayci M., Eren M.N., Yilmaz M., Kuloglu T., Gul E., Secen O. (2014). Decreased saliva/serum irisin concentrations in the acute myocardial infarction promising for being a new candidate biomarker for diagnosis of this pathology. Peptides.

[42] Lu J., Xiang G., Liu M., Mei W., Xiang L., Dong J. (2015). Irisin protects against endothelial injury and ameliorates atherosclerosis in apolipoprotein E-Null diabetic mice. Atherosclerosis.

[43] Lörchner H., Pöling J., Gajawada P., Hou Y., Polyakova V., Kostin S., Adrian-Segarra J.M., Boettger T., Wietelmann A., Warnecke H. (2015). Myocardial Healing Requires Reg3beta-Dependent Accumulation of Macrophages in the Ischemic Heart. Nat. Med..

[44] Luo Y., Qiao X., Ma Y., Deng H., Xu C.C., Xu L. (2020). Disordered metabolism in mice lacking irisin. Sci. Rep..

[45] Taylor C.J., Ordóñez-Mena J.M., Roalfe A.K., Lay-Flurrie S., Jones N., Marshall T., Hobbs F.D.R. (2019). Trends in survival after a diagnosis of heart failure in the United Kingdom 2000–2017: Population based cohort study. BMJ.

[46] Zhou B., Tian R. (2018). Mitochondrial dysfunction in pathophysiology of heart failure. J. Clin. Investig..

[47] Porter G.A., Hom J.R., Hoffman D.L., Quintanilla R.A., Bentley K.L.D.M., Sheu S.-S. (2011). Bioenergetics, mitochondria, and cardiac myocyte differentiation. Prog. Pediatr. Cardiol..

[48] Rosca M., Hoppel C.L. (2013). Mitochondrial dysfunction in heart failure. Heart Fail. Rev..

[49] Zhao Q., Sun Q., Zhou L., Liu K., Jiao K. (2019). Complex Regulation of Mitochondrial Function during Cardiac Development. J. Am. Heart Assoc..

[50] Menshikova E.V., Ritov V.B., Fairfull L., Ferrell R.E., Kelley D.E., Goodpaster B.H. (2006). Effects of Exercise on Mitochondrial Content and Function in Aging Human Skeletal Muscle. J. Gerontol. Ser. A Boil. Sci. Med. Sci..

[51] White A.T., Schenk S. (2012). NAD+/NADH and skeletal muscle mitochondrial adaptations to exercise. Am. J. Physiol. Metab..

[52] Puigserver P., Spiegelman B.M. (2003). Peroxisome Proliferator-Activated Receptor-Gamma Coactivator 1 Alpha (Pgc-1 Alpha): Transcriptional Coactivator and Metabolic Regulator. Endocr. Rev..

[53] Gureev A.P., Shaforostova E.A., Popov V.N. (2019). Regulation of Mitochondrial Biogenesis as a Way for Active Longevity: Interaction Between the Nrf2 and PGC-1α Signaling Pathways. Front. Genet..

[54] Riehle C., Abel E.D. (2012). PGC-1 Proteins and Heart Failure. Trends Cardiovasc. Med..

[55] Oka S.-I., Sabry A.D., Cawley K.M., Warren J.S. (2020). Multiple Levels of PGC-1α Dysregulation in Heart Failure. Front. Cardiovasc. Med..

[56] Wang S., Fu C., Wang H., Shi Y., Xu X., Chen J., Song X., Sun K., Wang J., Fan X. (2007). Polymorphisms of the Peroxisome Proliferator-Activated Receptor-Gamma Coactivator-1alpha Gene Are Associated with Hypertrophic Cardiomyopathy and Not with Hypertension Hypertrophy. Clin. Chem. Lab. Med..

[57] Arhire L.I., Mihalache L., Covasa M. (2019). Irisin: A Hope in Understanding and Managing Obesity and Metabolic Syndrome. Front. Endocrinol..

[58] Perakakis N., Triantafyllou G.A., Fernández-Real J.M., Huh J.Y., Park K.H., Seufert J., Mantzoros C.S. (2017). Physiology and role of irisin in glucose homeostasis. Nat. Rev. Endocrinol..

[59] Rosen E.D., Spiegelman B.M. (2006). Adipocytes as regulators of energy balance and glucose homeostasis. Nat. Cell Biol..

[60] Xiong X.-Q., Chen D., Sun H.-J., Ding L., Wang J.-J., Chen Q., Li Y.-H., Zhou Y.-B., Han Y., Zhang F. (2015). FNDC5 overexpression and irisin ameliorate glucose/lipid metabolic derangements and enhance lipolysis in obesity. Biochim. Biophys. Acta Mol. Basis Dis..

[61] Kurdiova T., Balaz M., Vician M., Maderova D., Vlcek M., Valkovic L., Srbecky M., Imrich R., Kyselovicova O., Belan V. (2014). Effects of Obesity, Diabetes and Exercise on Fndc5 Gene Expression and Irisin Release in Human Skeletal Muscle and Adipose Tissue: In Vivo and In Vitro Studies. J. Physiol..

[62] Vaughan R.A., Gannon N.P., Barberena M.A., Garcia-Smith R., Bisoffi M., Mermier C.M., Conn C.A., Trujillo K.A. (2014). Characterization of the Metabolic Effects of Irisin on Skeletal Muscle In Vitro. Diabetes Obes. Metab..

[63] Belviranlı M., Okudan N. (2018). Exercise training increases cardiac, hepatic and circulating levels of brain-derived neurotrophic factor and irisin in young and aged rats. Horm. Mol. Biol. Clin. Investig..

[64] Matsuo Y., Gleitsmann K., Mangner N., Werner S., Fischer T., Bowen T.S., Kricke A., Matsumoto Y., Kurabayashi M., Schuler G. (2015). Fibronectin type III domain containing 5 expression in skeletal muscle in chronic heart failure-relevance of inflammatory cytokines. J. Cachex Sarcopenia Muscle.

[65] Peng Q., Ding R., Wang X., Yang P., Jiang F., Chen X. (2021). Effect of Irisin on Pressure Overload–Induced Cardiac Remodeling. Arch. Med. Res..

[66] Peng Q., Wang X., Wu K., Liu K., Wang S., Chen X. (2017). Irisin attenuates H_2_O_2_-induced apoptosis in cardiomyocytes via microRNA-19b/AKT/mTOR signaling pathway. Int. J. Clin. Exp. Pathol..

[67] Li R., Wang X., Wu S., Wu Y., Chen H., Xin J., Li H., Lan J., Xue K., Li X. (2019). Irisin ameliorates angiotensin II-induced cardiomyocyte apoptosis through autophagy. J. Cell. Physiol..

[68] Lecker S.H., Zavin A., Cao P., Arena R., Allsup K., Daniels K.M., Joseph J., Schulze P.C., Forman D.E. (2012). Expression of the Irisin Precursor FNDC5 in Skeletal Muscle Correlates with Aerobic Exercise Performance in Patients with Heart Failure. Circ. Heart Fail..

[69] Sobieszek G., Powrózek T., Mazurek M., Skwarek-Dziekanowska A., Małecka-Massalska T. (2020). Electrical and Hormonal Biomarkers in Cachectic Elderly Women with Chronic Heart Failure. J. Clin. Med..

[70] Mansur A.J. (2018). Adropin and Irisin in Patients with Cardiac Cachexia. Arq. Bras. Cardiol..

[71] De Keulenaer G.W., Brutsaert D.L. (2011). Systolic and Diastolic Heart Failure Are Overlapping Phenotypes within the Heart Failure Spectrum. Circulation.

[72] Silvestrini A., Bruno C., Vergani E., Venuti A., Favuzzi A.M.R., Guidi F., Nicolotti N., Meucci E., Mordente A., Mancini A. (2019). Circulating irisin levels in heart failure with preserved or reduced ejection fraction: A pilot study. PLoS ONE.

[73] Rosendorff C., Lackland D.T., Allison M., Aronow W.S., Black H.R., Blumenthal R.S., Cannon C.P., De Lemos J.A., Elliott W.J., Findeiss L. (2015). Treatment of Hypertension in Patients with Coronary Artery Disease: A Scientific Statement from the American Heart Association, American College of Cardiology, and American Society of Hypertension. Circulation.

[74] Lamprea-Montealegre J.A., Zelnick L., Hall Y.N., Bansal N., De Boer I.H. (2018). Prevalence of Hypertension and Cardiovascular Risk According to Blood Pressure Thresholds Used for Diagnosis. Hypertension.

[75] Campbell N.R., Burgess E., Choi B.C., Taylor G., Wilson E., Cléroux J., Fodor J.G., Leiter L.A., Spence D. (1999). Lifestyle modifications to prevent and control hypertension. 1. Methods and an overview of the Canadian recommendations. Canadian Hypertension Society, Canadian Coalition for High Blood Pressure Prevention and Control, Laboratory Centre for Disease Control at Health Canada, Heart and Stroke Foundation of Canada. Can. Med. Assoc. J..

[76] Park K.H., Zaichenko L., Brinkoetter M., Thakkar B., Sahin-Efe A., Joung K.E., Tsoukas M., Geladari E.V., Huh J.Y., Dincer F. (2013). Circulating Irisin in Relation to Insulin Resistance and the Metabolic Syndrome. J. Clin. Endocrinol. Metab..

[77] Zhang L.-J., Xie Q., Tang C.-S., Zhang A.-H. (2017). Expressions of irisin and urotensin II and their relationships with blood pressure in patients with preeclampsia. Clin. Exp. Hypertens..

[78] Çelik H.T., Akkaya N., Erdamar H., Gok S., Kazanci F., DemirCelik B., Cakmak M., Yigitoglu R. (2015). The Effects of Valsartan and Amlodipine on the Levels of Irisin, Adropin, and Perilipin. Clin. Lab..

[79] Jiang M., Wan F., Wang F., Wu Q. (2015). Irisin relaxes mouse mesenteric arteries through endothelium-dependent and endothelium-independent mechanisms. Biochem. Biophys. Res. Commun..

[80] Zhang W., Chang L., Zhang C., Zhang R., Li Z., Chai B., Li J., Chen E., Mulholland M. (2015). Central and Peripheral Irisin Differentially Regulate Blood Pressure. Cardiovasc. Drugs Ther..

[81] Carling D., Viollet B. (2015). Beyond Energy Homeostasis: The Expanding Role of AMP-Activated Protein Kinase in Regulating Metabolism. Cell Metab..

[82] Han F., Zhang S., Hou N., Wang D., Sun X. (2015). Irisin Improves Endothelial Function in Obese Mice through the Ampk-Enos Pathway. Am. J. Physiol. Heart Circ. Physiol..

[83] Wu J., Spiegelman B.M. (2014). Irisin ERKs the Fat. Diabetes.

[84] Levy D., Larson M.G., Vasan R.S., Kannel W.B., Ho K.K. (1996). The progression from hypertension to congestive heart failure. JAMA.

[85] Delezie J., Handschin C. (2018). Endocrine Crosstalk Between Skeletal Muscle and the Brain. Front. Neurol..

[86] Li R.L., Wu S.S., Wu Y., Wang X.X., Chen H.Y., Xin J.J., Li H., Lan J., Xue K.Y., Li X. (2018). Irisin Alleviates Pressure Overload-Induced Cardiac Hypertrophy by Inducing Protective Autophagy via Mtor-Independent Activation of the Ampk-Ulk1 Pathway. J. Mol. Cell. Cardiol..

[87] Huo C.-J., Yu X.-J., Sun Y.-J., Li H.-B., Su Q., Bai J., Li Y., Liu K.-L., Qi J., Zhou S.-W. (2020). Irisin lowers blood pressure by activating the Nrf2 signaling pathway in the hypothalamic paraventricular nucleus of spontaneously hypertensive rats. Toxicol. Appl. Pharmacol..

[88] Sun N., Chen Y., Fan Y., Chang J., Gao X., Zhao Y., Sun H., Wang Z., Gu X., Tian J. (2021). Plasma irisin levels are associated with hemodynamic and clinical outcome in idiopathic pulmonary arterial hypertension patients. Intern. Emerg. Med..

[89] Petrie J., Guzik T.J., Touyz R.M. (2018). Diabetes, Hypertension, and Cardiovascular Disease: Clinical Insights and Vascular Mechanisms. Can. J. Cardiol..

[90] Liu X., Mujahid H., Rong B., Lu Q.-H., Zhang W., Li P., Li N., Liang E.-S., Wang Q., Tang D.-Q. (2017). Irisin inhibits high glucose-induced endothelial-to-mesenchymal transition and exerts a dose-dependent bidirectional effect on diabetic cardiomyopathy. J. Cell. Mol. Med..

